# Engineering matrix-embedded dual-drug eluting biodegradable surgical sutures for prophylaxis against surgical site infections

**DOI:** 10.1039/d6ra01817c

**Published:** 2026-06-01

**Authors:** Garba Mohammed Khalid, Neel Anil Chaudhary, Nura Hassan, Lubabah Rahman, Shahnaz Kashif, Gunjan V. Jahakar, Mouhamad Khoder

**Affiliations:** a School of Life Sciences, Pharmacy and Chemistry, Faculty of Health, Science, Social Care and Education, Kingston University Penrhyn Road Kingston upon Thames KT1 2EE UK; b School of Pharmacy, Newcastle University Newcastle upon Tyne NE1 7RU UK khalid.garba@newcastle.ac.uk

## Abstract

Despite advances in aseptic technique and conventional antimicrobial prophylaxis, surgical site infections (SSIs) remain a leading cause of postoperative morbidity, mortality, and healthcare costs globally. Traditional sutures merely approximate tissue and can harbour microorganisms that drive the emergence of multidrug-resistant pathogens. Thus, SSIs contribute significantly to the spread of antimicrobial resistance (AMR). To address this dual challenge, this study focuses on developing and characterize biodegradable, multi-drug-eluting sutures capable of localised delivery of two therapeutic agents to prevent infection and promote wound healing. Two clinically relevant therapeutic agents, levofloxacin (broad-spectrum antibiotic) and ibuprofen (an anti-inflammatory), were incorporated into biodegradable polycaprolactone (PCL) polymer. Placebo (F1), levofloxacin-only (F2), and dual-drug (F3) biodegradable sutures conforming to the USP 2–0 size specification were successfully fabricated using solvent-free hot-melt extrusion technology. Solid-state analyses (DSC and PXRD), as well as FTIR and Raman spectroscopy, confirmed that the drugs were amorphous and molecularly dispersed in the suture. *In vitro* drug release was diffusion-controlled and best fitted by the Korsmeyer–Peppas model. Mechanical properties remained suitable, with Young's modulus and elongation at break unaffected by drug loading, and tensile strength ranging from 88.3 ± 12.9 MPa (F1) to 69.5 ± 7.8 MPa (F3). This work demonstrates the feasibility of hot-melt extrusion as a solvent-free and green technique to produce sustained-release, multi-drug-eluting sutures with acceptable handling properties for clinical application.

## Abstract 2

This work engineered biodegradable drug-eluting sutures *via* solvent-free hot-melt extrusion, enabling immediate and sustained levofloxacin and ibuprofen release to prevent surgical site infections while controlling inflammation.

## Graphical abstract

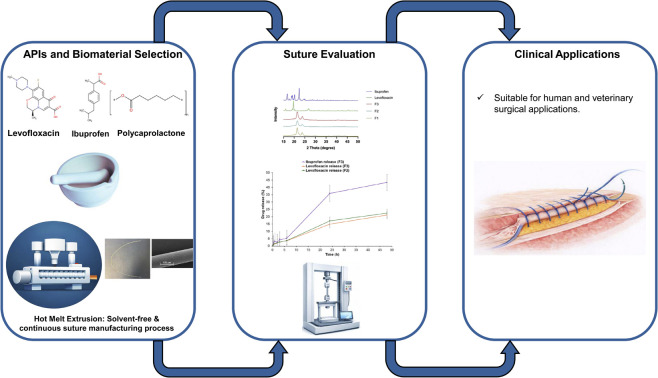

## 1. Introduction

Surgical sutures, commonly referred to as ‘stitches,’ are medical devices used to hold body tissues together.^1^ One of the most frequent healthcare-associated infections is surgical site infection (SSI), which happens when microorganisms enter the surgical site and proliferate in the tissues. SSIs continue to rank among the most prevalent infections linked to healthcare globally, impacting 2–5% of patients in high-income countries and up to 12% of surgical patients in low- and middle-income countries.^2^ According to a meta-analysis by Fan *et al.*, the pooled incidence rate of SSI was 4.5% in mainland China, 1.9% in the United States, 1.6% in Germany, 1.4% in England, 1.6% in France, and 2.0% in Portugal-substantially lower than the 11.8% average reported in developing countries, indicating improved infection control in developed countries.^3^ SSIs raise hospital stays, increase morbidity, and significantly raise healthcare expenses.^4^ SSIs are primarily caused by microbial pathogens, including Gram-negative bacteria such as *Escherichia coli*, *Pseudomonas aeruginosa*, *Acinetobacter* species, and *Enterococcus* species, as well as Gram-positive bacteria like *Staphylococcus aureus,* both methicillin-resistant and methicillin-sensitive strains.^5^

Antimicrobial-coated sutures, like Vicryl Plus® coated with triclosan, were created to address the problem of SSIs and have demonstrated lower rates of SSIs in certain clinical trials.^6^ Long-term efficacy has been questioned, though, due to triclosan resistance and its narrow range of action. Additionally, these sutures do not have anti-inflammatory properties, which are essential for lowering postoperative pain, swelling, and tissue trauma-related delayed healing.^6^

To provide dual protection against infection and excessive inflammation, the next-generation suture should ideally combine mechanical integrity with the controlled release of antimicrobial and anti-inflammatory agents. Thus, multifunctional sutures that can handle the problems of microbial infection and postoperative inflammation can be created by combining therapeutic agents like ibuprofen (NSAIDs), and levofloxacin, a broad-spectrum fluoroquinolone antibiotic.^7,8^

Traditionally, drugs can be incorporated into sutures using a variety of manufacturing techniques, each with unique advantages and limitations. For instance, electrospinning permits processing at lower temperatures, making it appropriate for thermolabile drugs, and allows control of suture diameter in a single step. However, the use of electric fields and organic solvents can damage labile APIs and decrease mechanical strength at higher drug loads. Coating methods like layer-by-layer or dip-coating are simple to use and can improve or preserve tensile strength even when there is a high drug content. However, these methods require the total elimination of organic solvents used in processing. By adjusting grafting parameters, grafting provides control over drug release profiles and surface chemistry. Nevertheless, residual solvents or monomers may adversely affect mechanical properties. Other techniques, such as supercritical CO_2_-assisted impregnation, are not appropriate for high-molecular-weight APIs and may significantly alter the microstructure of sutures. In contrast to the said techniques, hot melt extrusion (HME) is a solvent-free method that enhances drug solubility, dissolution rate, and subsequently, its bioavailability, by facilitating molecular-level dispersion of the drug within polymers, frequently resulting in amorphous solid dispersions.^9^ Additionally, the method enables exact control over formulation parameters like die design, temperature, and screw speed. Which can be modified to create devices or filaments with specific drug-release profiles, mechanical characteristics, and diameters.^9,10^ Similarly, advances in HME have propelled the technology in the development of drug-loaded filaments for pharmaceutical 3D printing and/or the design of such additive manufacturing platforms with the HME integrated into the 3D printing process, enabling solvent-free processing, controlled release profiles, dose personalisation, and scalable manufacturing of complex dosage forms.^11–13^

Advanced wound closure materials that can release one or more therapeutic agents under controlled conditions that actively prevent microbial colonisation, promote tissue repair, and offer mechanical support are urgently needed, as evidenced by the rising incidence of SSIs in both human and veterinary practice, including dirty conflict wounds. In this context, one of the promising options for next-generation biomedical devices is polycaprolactone (PCL), a semicrystalline, biodegradable, and biocompatible polyester. It is especially well-suited for long-term drug delivery applications due to its favourable mechanical qualities, slow rate of degradation, and capability for hot-melt extrusion at lower temperatures.

**Scheme 1:**
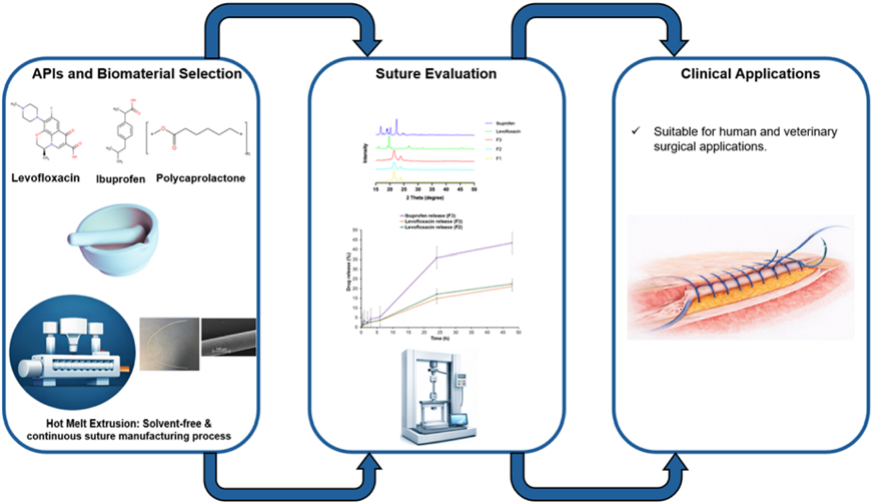
Schematic illustration of dual-drug eluting suture preparation, characterisations, and potential clinical applications.

The present study, therefore, aims to develop and assess biodegradable, multi-drug eluting surgical sutures intended to promote wound healing and prevent SSIs. PCL based sutures were fabricated by solvent-free HME and loaded with levofloxacin and ibuprofen. Placebo and drug-loaded sutures were characterised for morphological integrity, physicochemical and mechanical properties, solid-state, drug content and *in vitro* drug release, Scheme 1. The key gap addressed in our study is the absence of multifunctional sutures capable of simultaneously controlling inflammation and providing sustained antimicrobial prophylaxis to prevent SSIs and limit antimicrobial resistance (AMR). The system delivers rapid anti-inflammatory release followed by prolonged antibacterial release, demonstrating a scalable, cleaner manufacturing approach and establishing a proof-of-concept for advanced wound-healing sutures.

## 2. Experimental

### 2.1 Materials

The active pharmaceutical ingredients, levofloxacin and ibuprofen, were supplied by TCI Ltd, United Kingdom and Fluorochem, United Kingdom, respectively. The polymer, polycaprolactone powder (PCL), (CAPA 6506, MW 50 000 g mol^−1^), was kindly gifted by Ingevity, United Kingdom. Chloroform (analytical grade, 99.8%), sodium phosphate monobasic (NaH_2_PO_4,_ MW 119.98 g mol^−1^), and sodium phosphate dibasic (Na_2_HPO_4_, MW 141.96 g mol^−1^) were purchased from Sigma Aldrich, United Kingdom. All other reagents were of analytical grade.

### 2.2 Methods

#### 2.2.1 Preparation of suture by hot melt extruder

Prior to melt mixing, PCL, levofloxacin, and Ibuprofen were evaluated for solid-state and thermal properties. They were then physically mixed and added to the HME barrel feeder. The HME screw was operated at 50 rpm. Extrusion was carried out using Noztek extruder (Noztek pro HME, UK) at 84 ± 1 °C. Suture filament was extruded through a die and manually pooled to achieve the target suture diameter, then rapidly solidified by a fan placed directly underneath the die. The placebo and drug-loaded suture formulations were made using the same conditions and kept in plastic bags at room temperature until further use. The compositions of the formulations are listed in Table 1.

**Table 1:** Compositions of placebo and drug-loaded sutures

Formulation	Polycaprolactone (% w/w)	Levofloxacin (% w/w)	Ibuprofen (% w/w)
F1	100.0	—	—
F2	90.0	10.0	—
F3	80.0	10.0	10.0

### 2.3 Characterisation of raw materials and suture

#### 2.3.1 Thermal analysis

##### 2.3.1.1 Differential scanning calorimetry (DSC)

DSC (DSC 25, Waters, USA), was used to characterise the thermal properties of the APIs, PCL and suture formulations. To ensure accuracy, high-purity aluminium standards were used for instrument calibration. Before the analysis, 3–5 mg of each sample was weighed and sealed in aluminium pans. The samples were equilibrated at 20 °C, then ramped to 250 °C at a heating rate of 10 °C min^−1^. Nitrogen was used as a purge gas at a flow rate of 50 mL min^−1^. Data was processed using Trios software.

##### 2.3.1.2 Thermogravimetric analysis (TGA)

Using Thermogravimetric Analysis (TA, TGA 550, Waters, USA) with Trios software, the formulation starting materials and sutures' thermal stability and decomposition behaviour were assessed. Samples 3–4 mg were placed in an aluminium pan. Nitrogen was used as a purge gas with a flow rate of 50 mL min^−1^. The samples were equilibrated at 30 °C, then ramped to 500 °C at 10 °C min^−1^. Percentage mass loss and/or onset temperature were calculated.

#### 2.3.2 Fourier transform infrared spectroscopy (FTIR)

A Nicolet Summit X fitted with EVEREST Attenuated Total Reflectance (ATR-FTIR) (Thermo Scientific, USA) was used to detect distinctive functional groups and validate drug incorporation in the biodegradable sutures using FTIR. Additionally, to provide reference peaks for comparative analysis, the spectra of the excipients and pure drugs were recorded. All samples were scanned over a wavelength range of 4000–650 cm^−1^ at a resolution of 4 cm^−1^ for 16 scans.

#### 2.3.3 Raman spectroscopy

An inVia Raman Microscope (Model: 23TN21, Renishaw, UK) was used for Raman analysis to examine molecular vibrations and verify drug incorporation. To find distinctive vibrational bands, the spectra of pure components, drug-loaded sutures, and neat PCL were obtained by placing the suture samples and illuminating them with a monochromatic laser light and analysing the Rayleigh scattering.

#### 2.3.4 X-ray diffraction analysis (XRD)

X-ray diffraction (XRD) analysis was performed on a Bruker-AXS Model D8-Advance powder diffractometer, using Ni-filtered Cu Ka radiation from a copper anode run at 40 kV and 20 mA. (*λ* = 1.5418 angstrom). A step scan over the range 10–60° 2*θ* was used, with intervals of 0.11 and a counting time of 8 s. The technique was used to identify the distinctive crystalline peaks of semicrystalline PCL and evaluate potential changes in crystallinity that may occur when ibuprofen and levofloxacin are added.

#### 2.3.5 Scanning electron microscopy (SEM)

A scanning electron microscope (SEM, EVO50, ZEISS, Germany) was used to analyse the surface morphology of the sutures, confirming their homogeneity and structural integrity. A smooth, continuous filament structure was visible in high-resolution imaging. Before imaging, the sutures were carbon-coated to increase electron beam conductivity and reduce charging effects. An accelerating voltage of 10 kV was used for the analysis.

#### 2.3.6 Mechanical properties

Tensile properties of the placebo and drug-loaded sutures were assessed using a texture analyser (TA.XT Express, D822-02, England). Placebo and drug-loaded sutures were equilibrated at 25 ± 1 °C for one week, and the test was performed with a 2.5 mm initial grip separation and at 10 mm s^−1^ crosshead speed until sample breaks. Tension was used in the test, and the following parameters were calculated:

Tensile strength – measured as the maximum amount of stress a suture can withstand before it breaks when being stretched or pulled, given by eqn (1).1
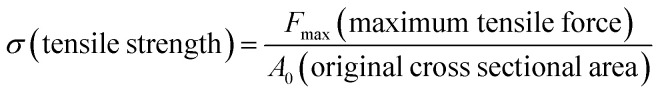


Elongation at break – is a measure of a material's ductility, indicating the maximum amount it can stretch before breaking, expressed by eqn (2).2
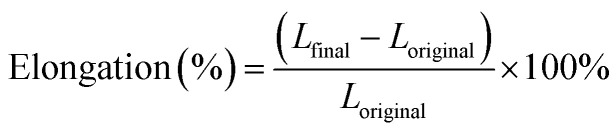


Young's module – measured as the suture's stiffness, calculated as the slope of the linear portion of the stress–strain curve. The result was expressed in force per unit area.

#### 2.3.7 Drug content determination

To verify successful drug incorporation and assess content uniformity, the drug content of the fabricated sutures was investigated. For levofloxacin, about 10 mg of F2 and F3 sutures were dissolved in acetonitrile and distilled water (20 : 80% v/v). The solution was then filtered *via* a 0.22 µm membrane filter (Millex, Merck Millipore Ltd, Ireland) into the vial and analysed using high-performance liquid chromatography with an ultraviolet detector (HPLC-UV) (Agilent Technologies 1260 infinity). A calibration curve was developed with levofloxacin in acetonitrile and water in the concentration range of 2.5 µL mL^−1^ to 150 µL mL^−1^. Chromatographic conditions: column: phenomenex 5 µm C18 column, 4.6 mm × 150 mm; detector wavelength: 294 nm; column temperature: 25 °C; flow rate: 1 mL min^−1^; injection volume: 10 µL; run time: 6.5 minutes. An isocratic condition was used with acetonitrile: phosphate buffer pH 3 (35 : 65% v/v) as the mobile phase. The phosphate buffer was prepared by dissolving 1.36 g of potassium dihydrogen phosphate in deionised water. The pH was adjusted to 3 using dilute hydrochloric acid.

For ibuprofen, about 10 mg of F3 suture was dissolved in a mixture of phosphate buffer (pH 6.8) and acetonitrile (65 : 35% v/v), filtered and analysed using HPLC with an ultraviolet detector (HPLC-UV) (Agilent Technologies 1260 infinity). Initially, a calibration curve was prepared using ibuprofen standard solutions in the mobile phase over the concentration range of 2.5 µg mL^−1^ to 150 µg mL^−1^. Chromatographic conditions: column: C18 reverse phase column: SphereClone ODS, 5 µm, 4.6 mm × 150 mm; detector wavelength: 222 nm, column temperature: 25 °C, flow rate: 0.7 mL min^−1^; injection volume: 10 µL; run time: 10 minutes. An isocratic condition was used with acetonitrile: phosphate buffer (65 : 35% v/v, pH 6.8) as the mobile phase.

#### 2.3.8 *In vitro* drug release

*In vitro* release of the drug-loaded sutures was performed to evaluate drug release from F2 and F3 formulations. A paddle-type dissolution apparatus (Copley Scientific, Model DIS 6000, Nottingham, UK) equipped with a NE4-COPD heater/circulator (230 V, 50/60 Hz, 240 W) was used to conduct the release studies. A 3 cm drug-loaded suture was placed in 150 mL of phosphate buffer saline (pH 7.3, to mimic human interstitial fluid and blood pH) in a USP paddle apparatus operated at 50 rpm and 37 °C. Three replicates were used for each formulation. Aliquots of 10 mL were withdrawn at predetermined intervals (15 min, 30 min, 1 h, 2 h, 3 h, 6 h, 24 h, and 48 h), and drug release was quantified by UV-vis Spectrophotometer (G105 UV-vis, Thermo Fisher Scientific, UK). The withdrawn sample was replaced with a fresh buffer to maintain sink conditions. Initially, both drugs were quantified using UV-vis spectroscopy, and separate calibration curves were constructed in the same dissolution medium in the concentration range of 2.5–150 µg mL^−1^ at 222 nm for ibuprofen and 289 nm for levofloxacin, respectively. For the dual-drug formulation (F3), the wavelength was switched between readings to avoid spectral overlap. Release data were fitted to zero-order, first-order, Higuchi, and Korsmeyer–Peppas models, with the Higuchi and Korsmeyer–Peppas models providing the best fit, indicating diffusion-controlled and anomalous transport.

#### 2.3.9 Statistical analysis

Microsoft Excel (Version 16.95.1, Build 25031528, 2025, USA) was used for statistical analyses including calculating mean, standard deviation, ANOVA, 95% Confidence intervals. The post-hoc method employed was Tukey's Honest Significant Difference (HSD) test, which ensures that observed differences in mechanical strength were not the result of random variation by offering a robust control of Type I error when performing multiple pairwise comparisons across the three formulations (F1, F2, and F3). Statistical significance difference is indicated when *p* ≤ 0.05. GraphPad Prism (Version 10.6.0, Build 796, 2025) was utilised for data processing and graphical design.

## 3. Results and discussion

### 3.1 Solid-state properties

**Fig. 1:**
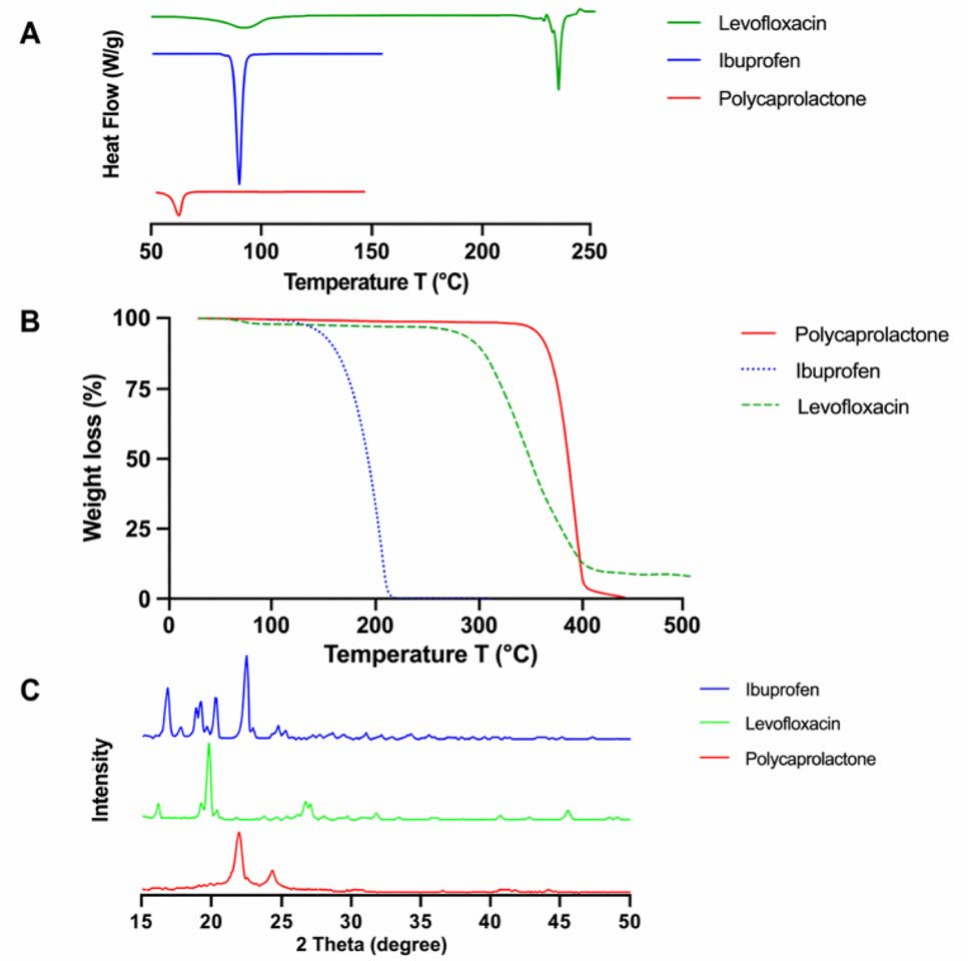
DSC thermograms of levofloxacin, ibuprofen, and PCL (A); TGA thermograms (B); and X-ray diffractograms (C).

The DSC analysis for each component showed unique endothermic melting curves, confirming their crystalline characteristics (Fig. 1A). Moreover, these measurements provided vital baseline pre-formulation data for appropriate HME processing.^14,15^

The melting endotherm of PCL appeared as a sharp peak at 61.9 °C.^16^ The melting point of PCL clearly lies within its known melting range of 58 to 65 °C, indicating its semi-crystalline nature. On the other hand, the melting point of ibuprofen appeared as a sharp peak at 75.5 °C.^17^ Similarly, the onset melting endotherm of levofloxacin appeared as a broad endothermic peak around 232 °C.^18^ In each case, the results observed corroborated the existing literature. Moreover, the DSC thermogram of levofloxacin shows a degradation peak around 250 °C, potentially due to strong intermolecular forces in high-melting-point compounds.^18^ Thus, partial thermal decomposition of levofloxacin may occur close to its melting point. Further, TGA analysis also supported the thermal stability of each material, with a single, distinct degradation step for PCL, with an onset temperature of around 342.7 °C (Fig. 1B), establishing its suitability for high-temperature processing. In contrast, ibuprofen and levofloxacin had earlier thermal degradation onset temperatures of 143.1 °C and 250.6 °C, respectively, which indicate their relatively lower thermal stability compared to the polymeric carrier.^19^ Altogether, the DSC and TGA data point toward a distinct thermal processing window where PCL can be melted while preserving the integrity of both active pharmaceutical compounds, underlining the importance of accurate temperature control during HME processing to avoid drug degradation while ensuring effective polymer softening and dispersion.

The X-ray diffractograms for pure drugs and polymer indicate the crystalline nature of these compounds in the solid state, matching the literature data. PCL had a characteristic peak, showing 2*θ* at 21.6° and 23.8° (Fig. 1C), corresponding to crystalline regions within an amorphous matrix, as reported for many polyesters.^20^ The diffractogram for levofloxacin indicated sharp, well-defined diagnostic 2*θ* peaks at 19.8° and 26.7°. These data suggest a highly ordered crystalline lattice, confirming its crystalline nature in the solid state.^21^ Similarly, the diffractogram for ibuprofen shows several sharp, intense fingerprint 2*θ* peaks at 16°, 18°, 20°, and 22°.^22^ Again, confirming the highly crystalline nature of ibuprofen, with long-range molecular order in the crystalline lattice, as reported in the literature for many crystalline compounds. These diffractograms confirm the identity of these compounds in the crystalline state, providing useful reference data for any changes in crystallinity, which could be induced by drug-loaded suture formation.

### 3.2 Raman and FTIR spectroscopy

**Fig. 2:**
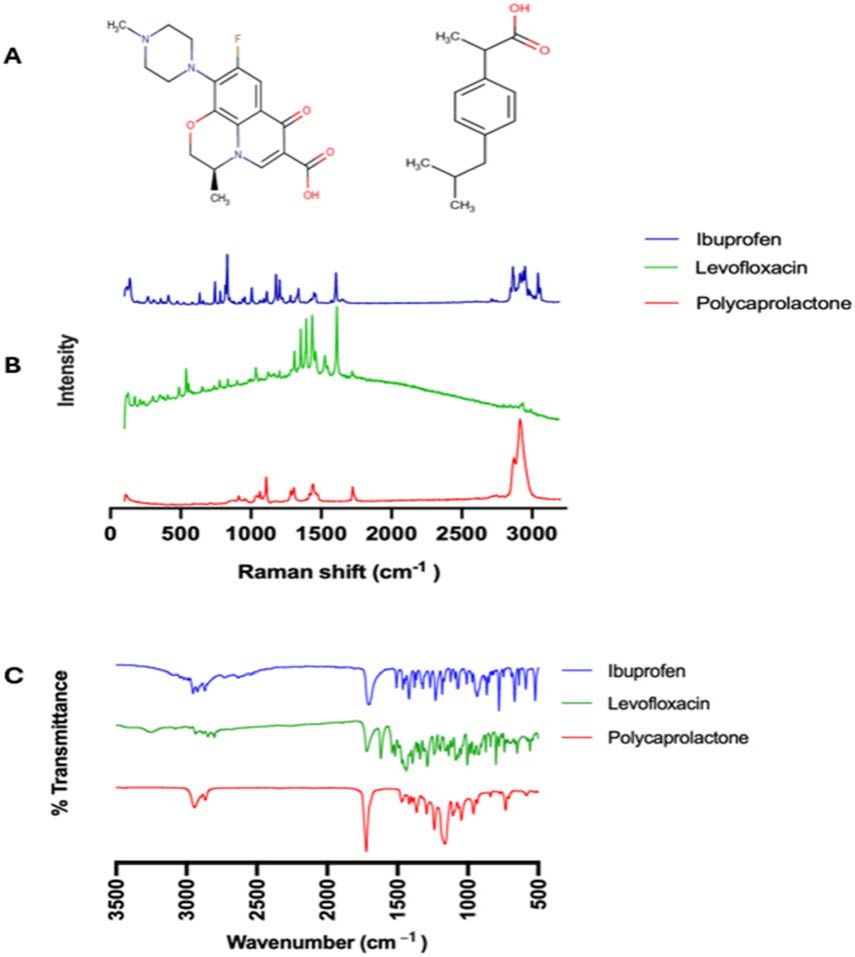
Chemical structures of levofloxacin (left), ibuprofen (right) (A); Raman spectral overlay of pure levofloxacin, ibuprofen, and PLC (B), and their corresponding FTIR spectral overlay (C).

Fig. 2A revealed the chemical structure of levofloxacin and ibuprofen to aid in discussing their chemical nature from the Raman and FTIR analyses. The Raman spectroscopic analysis of each compound depicts their distinct vibrational signatures, thereby verifying their chemical composition and functional groups, as well as providing a reliable reference point for the subsequent formulation comparisons. PCL (Fig. 2B) displays intense vibrational bands characteristic of its aliphatic polyester backbone, including a strong ester carbonyl (C=O) stretching vibration between 1715–1730 cm^−1^, methylene C–H stretching bands between 2865–2945 cm^−1^, as well as C–C skeletal stretching bands between 900–950 cm^−1^, which are characteristic of its semi-crystalline polymeric backbone.^23^ Ibuprofen was found to display distinct Raman spectral features characteristic of its aromatic and carboxylic acid functional groups, including a carboxylic acid C=O stretching band that overlaps with the PCL ester C=O stretching band between 1715–1730 cm^−1^, as well as aromatic C=C stretching bands between 1560–1580 cm^−1^, which are characteristic of its phenyl ring.^24^ Levofloxacin is readily distinguished from PCL and Ibuprofen based on its unique vibrational bands, including a C–F stretching band between 1200–1250 cm^−1^, as well as characteristic quinolone ring C=C and C=N stretching bands between 1600–1620 cm^−1^.^25^ The presence, location, and relative intensity of these characteristic Raman bands verify the structural integrity and chemical purity of each constituent, while at the same time offering critical spectral references to evaluate drug-polymer interaction, dispersion, and chemical alteration in the final processed hot-melt extruded formulations. Similarly, to complement the Raman spectroscopy, FTIR spectroscopy further confirmed the chemical characterisation and the presence of functional groups for each pure material. PCL displayed characteristic aliphatic C–H stretching vibrations at 2963 cm^−1^ and 2885 cm^−1^ (Fig. 2C), as well as a sharp, intense carbonyl (C=O) stretching band for ester groups at 1726 cm^−1^, which are consistent with its known polyester structure and literature values.^26^ The FTIR spectrum for levofloxacin displayed distinct absorption bands for this antibiotic material, including a broad O–H stretching for the carboxylic acid group at 3262 cm^−1^, a sharp intense carbonyl (C=O) stretching band at 1723 cm^−1^, as well as a sharp intense methyl (CH_3_) bending band at 1321 cm^−1^ and a sharp intense C–N stretching band at 1292 cm^−1^, which are consistent with its known quinolone and amine functionalities.^27^ Ibuprofen also displayed a sharp, intense carboxylic acid carbonyl (C=O) stretching band at 1752 cm^−1^, as well as aromatic C–C stretching vibrations in the 1200–1000 cm^−1^ region. Overall, the FTIR spectra of the components PCL, levofloxacin, and ibuprofen are clearly distinguishable, and there is strong agreement between them and their corresponding standard spectra.^28^

### 3.3 Morphological characteristics of the sutures

**Fig. 3:**
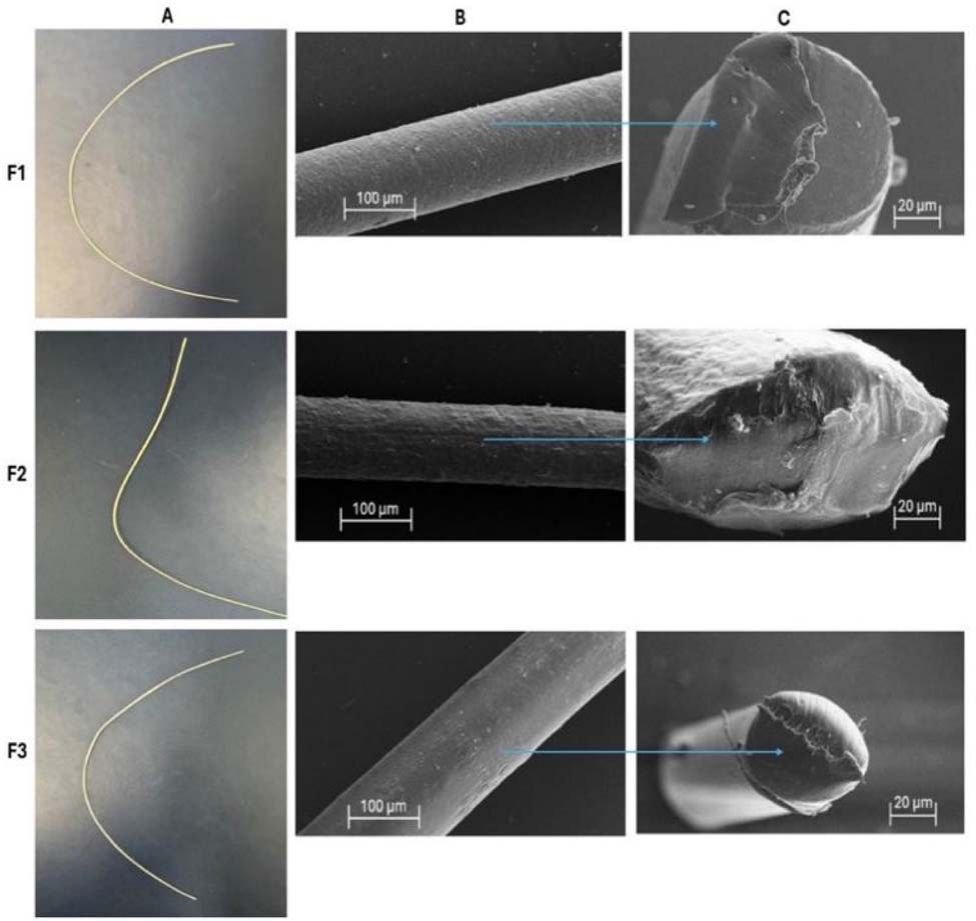
Placebo suture (F1), levofloxacin-only loaded suture (F2), levofloxacin and ibuprofen loaded suture (F3), physical appearance (A); SEM suture surface view (B); SEM suture cross-sectional view (C).

Morphological characterisation was carried out to assess the microscopic uniformity, surface topography, and dimensional similarity of the extruded sutures, as these are critical for mechanical properties and controlled drug delivery. Visual observation of the filaments (Fig. 3A) confirmed a uniform and smooth appearance for placebo and drug-loaded formulations, which had good surface continuity and uniform yellowish-white colouration, indicating good extrusion conditions and good material flow characteristics. The placebo and drug-loaded sutures exhibited mean filament diameters corresponding to a grade 2–0 suture according to USP specifications (Table 2).^29^

**Table 2:** Physicochemical and mechanical properties of placebo and drug-loaded sutures

Formulation	Diameter (mm)	USP classification	Tensile strength (MPa)	Young's modulus (MPa)	Elongation at break (%)	Drug content (%)
F1	0.327 ± 0.04	2-0	88.36 ± 12.93	11.19 ± 4.68	416.75 ± 120.78	—
F2	0.308 ± 0.05	2-0	69.51 ± 14.87	14.89 ± 5.91	406.66 ± 117.31	100.00 ± 1.36^a^
F3	0.314 ± 0.05	2-0	67.33 ± 7.85	16.02 ± 6.35	369.39 ± 107.11	100.00 ± 0.22^a^
						100.00 ± 1.42^b^

a Levofloxacin.

b Ibuprofen.

Furthermore, SEM offered additional information on the microstructure of the sutures regarding the surface morphology and homogeneity of the internal and exterior structures of the sutures. All formulations, *i.e.*, F1, F2, and F3, exhibited smooth and continuous surface morphology (Fig. 3B) without any fractures, cracks, or surface defects. Moreover, the cross-sectional view (Fig. 3C), also showed a compact internal structure of the suture matrix, indicating homogeneity and structural integrity of the fabricated sutures. The placebo suture, *i.e.*, F1, exhibited a highly uniform surface morphology, indicating the characteristic of semicrystalline PCL.^19^ On the other hand, the levofloxacin-only loaded suture, *i.e.*, F2, exhibited a slightly rough surface relative to F1, which may be attributed to drug dispersion in the polymer at low extrusion temperature.^30^ Most importantly, the dual drug-loaded suture, *i.e.*, F3, exhibited homogeneous surface along the entire length of the suture, *i.e.*, without any evidence of drug agglomeration, drug-polymer separation, or delamination.^31^ The texturing of the surface of drug-containing sutures may enhance sustained drug release, while their structural integrity indicates that drug incorporation has not compromised it, making them suitable for various applications in surgery and biomedicine.

### 3.4 Solid-state and chemical properties of suture

**Fig. 4:**
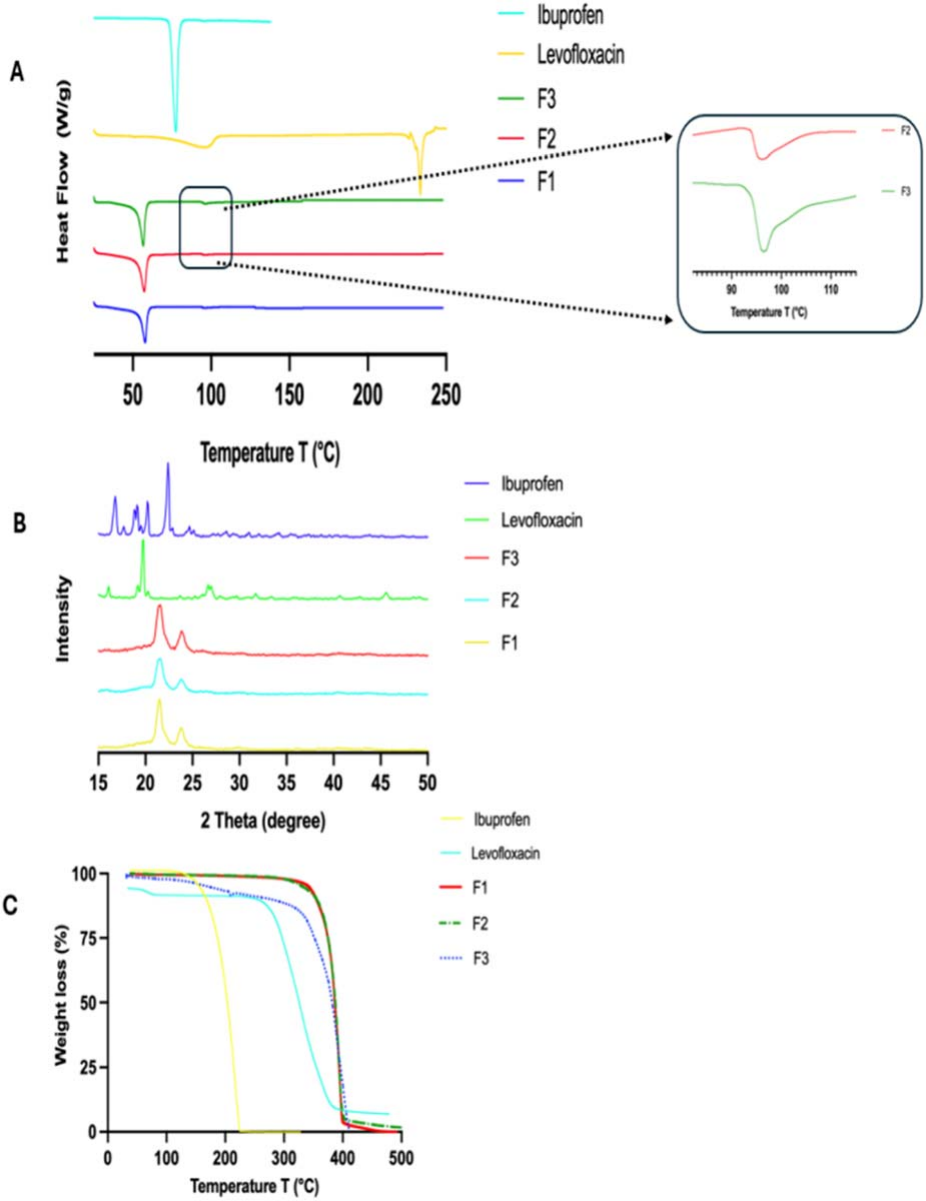
DSC thermograms of placebo and drug-loaded sutures, (A); their corresponding X-ray diffractograms (B); and TGA thermograms (C) compared to APIs.

To understand the influence and impact of thermal exposure during HME on the loaded APIs in the sutures, DSC, PXRD, TGA, Raman, and FTIR analyses were performed on the placebo and drug-loaded sutures. DSC analysis of the formulated sutures showed changes in the thermal behaviour of the incorporated drugs. The melting endotherms of F1 showed a sharp peak at about 57.58 °C (Fig. 4A). This melting point corresponds to the melting point of PCL. The melting endotherms of ibuprofen and levofloxacin were absent in the suture formulations containing the drugs F2 and F3. Only the melting endotherm of PCL remained for all formulations, with slight changes in the enthalpy. The enthalpy changes suggest slight changes in the crystallinity of PCL due to the addition of drugs. To confirm this hypothesis, the thermograms of formulations F2 and F3 were re-plotted using a magnified view of the post-melting region of the thermogram from about 80 °C to 115 °C. The drug-loaded suture thermogram showed glass transition (Tg) events at 94 °C for F2 (levofloxacin only suture), and 92 °C for F3 (levofloxacin plus ibuprofen loaded suture). The complete absence of melting peaks and appearance of Tg in the drug-loaded sutures (F2 and F3) suggests conversion of both drugs into an amorphous form and molecularly dispersed in the polymer during the HME process. This phenomenon of inhibition in the process of crystallisation is typically observed in the case of drug-polymer combinations due to the homogeneous distribution of the drug particles and the intermolecular interactions. Such an effect is also advantageous since the amorphization may improve the process of dissolution and drug release.^32,33^ To confirm these observations, the DSC results are used as complementary to the PXRD results.

The PXRD pattern of the placebo suture sample F1, as depicted in Fig. 4B, indicates the presence of two intense diffraction peaks at 21.5° and 23.9°, corresponding to the characteristic peaks of the orthorhombic crystal structure of PCL, which correspond to the (110) and (200) planes,^33^ thus confirming that the semicrystalline nature of the PCL in the extruded suture sample has been retained. PXRD pattern of the levofloxacin-loaded suture sample F2 indicated the presence of only the characteristic peaks of PCL, with no additional intense peaks corresponding to the crystalline form of levofloxacin. Likewise, the PXRD pattern of the dual drug-loaded suture sample F3 indicated only the characteristic peaks of PCL, with no detectable peaks corresponding to the crystalline form of ibuprofen and/or levofloxacin. Thus, findings from the DSC corroborated well with the X-ray diffraction results and confirmed the loading of levofloxacin and ibuprofen as amorphous solid dispersions in the sutures matrix.

In addition, TGA analysis of the formulated sutures further proved the successful incorporation of drugs by showing differences in thermal stability between each formulated suture. The placebo suture (F1) had an onset of degradation at 335.28 °C (Fig. 4C), which is close to values given for pure PCL. This confirms that the polymer backbone has maintained a reasonable level of thermal stability after extrusion. However, it was noted that the levofloxacin-loaded suture (F2) had a lower onset of degradation at 324.31 °C, and that the onset of degradation in the dual-drug-loaded suture (F3) was about 161 °C lower, which confirms that the combined effect of ibuprofen and levofloxacin has disrupted the thermal stability of the drug-polymer combination. Although it has been noted that a reduction in thermal stability is a common problem in drug-loaded polymer matrices, it can be confirmed that it is well above the extrusion and physiological temperatures, making it suitable for biomedical applications. Moreover, it has been proven that drug incorporation has been successful while maintaining the structural integrity necessary for drug delivery.

**Fig. 5:**
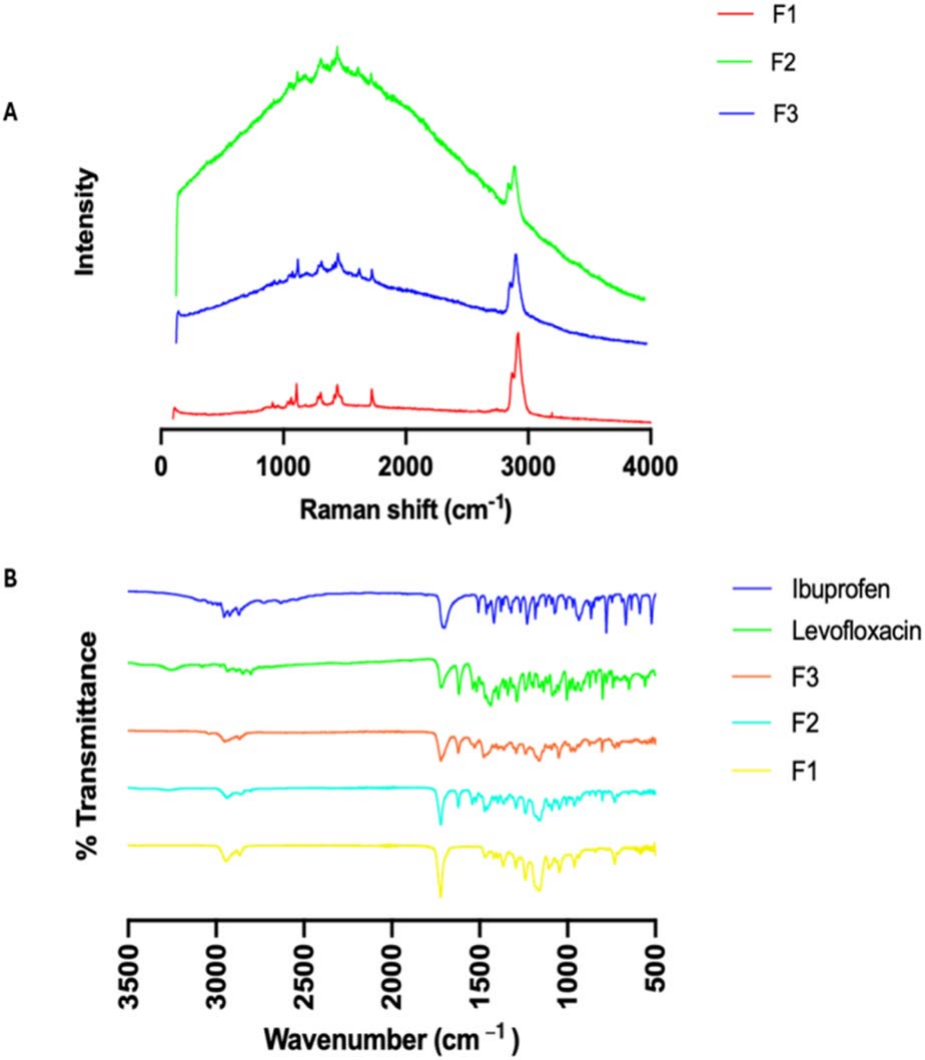
Raman spectral overlay of placebo and drug-loaded sutures (A), and their corresponding FTIR spectral overlay (B).

The Raman spectra for all fabricated suture formulations (F1, F2, and F3) have shown distinct vibrational bands, confirming the successful incorporation of ibuprofen, and levofloxacin in the polymer matrix, Fig. 5A. Bands for PCL, such as the ester carbonyl stretching vibration at 1715–1730 cm^−1^, methylene C–H stretching vibrations at 2865–2945 cm^−1^, and C–C skeletal vibrations at 900–950 cm^−1^, have been observed for all formulations, which confirm that the structural integrity of the polymer is maintained even after extrusion. In addition, for drug-loaded formulations, diagnostic bands for levofloxacin, such as the C–F stretching vibration at 1200–1250 cm^−1^ and quinolone ring C=C/C=N stretching vibrations at 1600–1620 cm^−1^ have been clearly observed in the Raman spectra, which confirm the successful incorporation of this API in the polymer matrix. Bands for ibuprofen have also been observed in terms of broadening in the carbonyl stretching region at 1715–1730 cm^−1^ and aromatic C=C stretching vibrations at 1560–1580 cm^−1^, as well as aromatic C–H stretching vibrations at 2968 cm^−1^. These Raman bands confirm that both APIs have maintained their characteristic vibrational identities and are homogenously distributed in the polymer matrix.

Similarly, FTIR spectroscopy offered definitive evidence for the simultaneous incorporation of two drugs and their chemical integrity in the PCL suture matrix. The FTIR spectrum of the dual-drug-loaded formulation (F3) (Fig. 5B), when compared to the pure PCL and the individual APIs (ibuprofen and levofloxacin), showed the presence of all the characteristic polymer absorption peaks, such as aliphatic C–H stretching vibrations at 2965–2968 cm^−1^ and 2885 cm^−1^, along with a strong ester carbonyl (C=O) peak at 1726–1728 cm^−1^, thus establishing the integrity of the PCL polymer after formulation. This carbonyl region was overlapped by the carboxylic acid C=O stretching of ibuprofen, leading to a slight increase in the band intensity. The presence of levofloxacin was also established by a broad O–H stretching band at 3260–3270 cm^−1^, along with peaks at 1245 cm^−1^ (C–N stretching) and 1082 cm^−1^ (C–F stretching). Additionally, ibuprofen-related aromatic peaks were seen from bands in the 1200–1000 cm^−1^ region and aromatic C=C stretching at 1560–1580 cm^−1^.

Overall, the retention and overlap of these diagnostic bands verify that both APIs were chemically intact and compatible within the PCL matrix, thus confirming the successful incorporation of both drugs within the suture.

### 3.5 Tensile properties

**Fig. 6:**
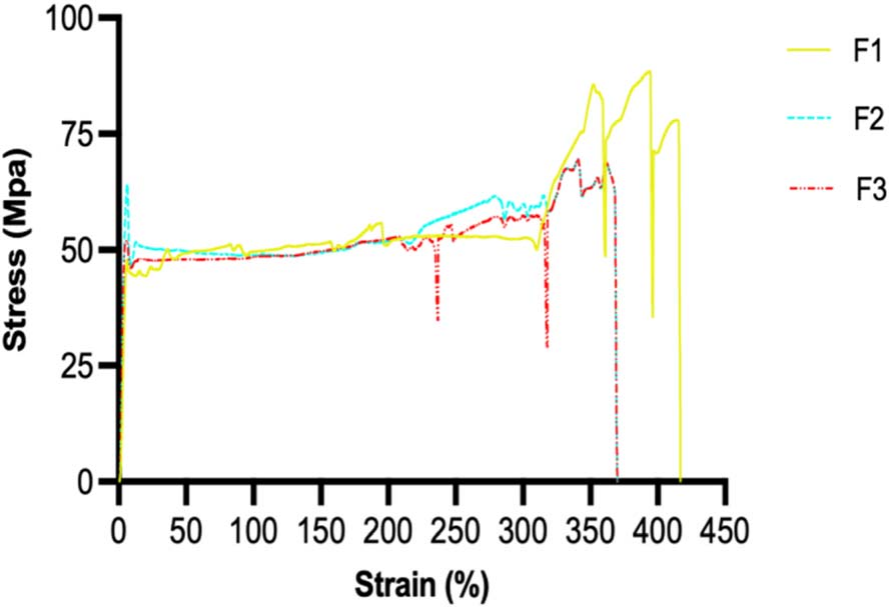
Stress–strain curves for placebo and drug-loaded sutures.

Tensile properties ensure sutures withstand tissue tension during healing and body movement, preventing breakage, dehiscence, and necrosis by maintaining proper wound edge apposition.^34^ The tensile properties result revealed that F1 had the strongest mechanical profile with tensile strength of 88.36 ± 12.93 MPa, Table 2, Fig. 6. As anticipated, PCL is a semicrystalline polymer with high stiffness and load-bearing capacity. The Young's modulus and elongation at break of the placebo suture relative to drug-loaded ones demonstrated intrinsic ductility and PCL's capacity to experience substantial deformation before failing. The effect of drug loading on tensile strength and suture elongation at break was statistically significant (*p* = 0.0133) and (*p* = 0.0786), respectively. The Young's modulus, however, shows no difference between the placebo and drug-loaded sutures (*p* = 0.4233).

Due to the small sample size (*n* = 3), not pairwise comparisons survived Bonferroni/Holm adjustment (*α* = 0.0167), although post-hoc Welch *t*-tests showed that the F1 *vs.* F2 contrast approached significance (*p* = 0.041). However, the overall statistical results suggest that the impact of drug incorporation on suture strength varies with formulation. The tensile strength of F2 was moderately reduced (95% CI: 69.46–69.57) relative to F1. However, pairwise comparisons failed to achieve significance following correction. F2 surprisingly displayed a higher Young's modulus of 14.89 ± 5.91 MPa (95% CI: 3.55–26.11) in terms of stiffness; however, ANOVA for modulus did not show a significant difference between groups (*p* = 0.4233). At break, the elongation revealed no discernible difference from F1 (*p* = 0.0786). According to these results, levofloxacin decreased strength and disturbed crystalline packing, but it also decreased chain mobility in the elastic region, increasing stiffness without appreciably reducing ductility.

The mechanical profile of F3 was the weakest, with a tensile strength of 67.33 ± 7.85 MPa (95% CI: 41.59–79.77). Tensile strength differences were found to be significant (*p* = 0.0133) by ANOVA; however, post-hoc tests once more revealed no pairwise significance following correction. F3 had the highest Young's modulus of 16.02 ± 6.35 MPa (95% CI: 13.61–18.43); however, ANOVA revealed no significant group difference (*p* = 0.4233). Elongation at break decreased to 369.39 ± 107.11% (95% CI: 140.34–474.59), but this decrease was not considered significant by ANOVA (*p* = 0.0786). According to these results, the polymer microstructure was more severely disrupted by the simultaneous addition of levofloxacin and ibuprofen, resulting in embrittlement and decreased ductility, though not to a statistically significant extent given the current sample size.

The observed reduction in ductility (elongation at break from 416.75% for F1 to 369.39% for F3) warrants consideration for clinical translation. However, it's noted that even the lowest elongation value (369%) remains well above the requirements for most surgical applications, where typical tissue elongation rarely exceeds 30–50%. The tensile strength of F3 (67.33 MPa) remains comparable to that of commercially available absorbable sutures of equivalent USP size. Thus, despite moderate embrittlement because of the drug loading, the mechanical performance remained suitable for soft tissue approximation. Mechanical properties of biodegradable sutures may gradually decrease during drug release due to pore generation following API diffusion and progressive polymer hydrolysis. However, because PCL undergoes relatively slow degradation, clinically relevant tensile support is expected to be retained during the critical early wound-healing phase.^46^

### 3.6 Drug content

Both the single-drug-loaded (F2) and the dual-drug-loaded (F3) sutures had their drug content ascertained by HPLC analysis. Using different chromatographic conditions, the retention times for both drugs were 4 min and 2.5 min for levofloxacin and ibuprofen, respectively. The drug content in both F2 and F3 for levofloxacin alone and levofloxacin and ibuprofen sutures was found to be within acceptable limit for drug content, *i.e.*, L1 = ±10% (Table 2). Moreover, there was no additional peak from the HPLC chromatogram to suggest any impurities and/or degradation products generated because of the HME process. Thus, confirming the suitability of HME as an enabling technology for loading these drugs in the biodegradable sutures with PCL as the carrier polymer.

### 3.7 *In vitro* drug release

**Fig. 7:**
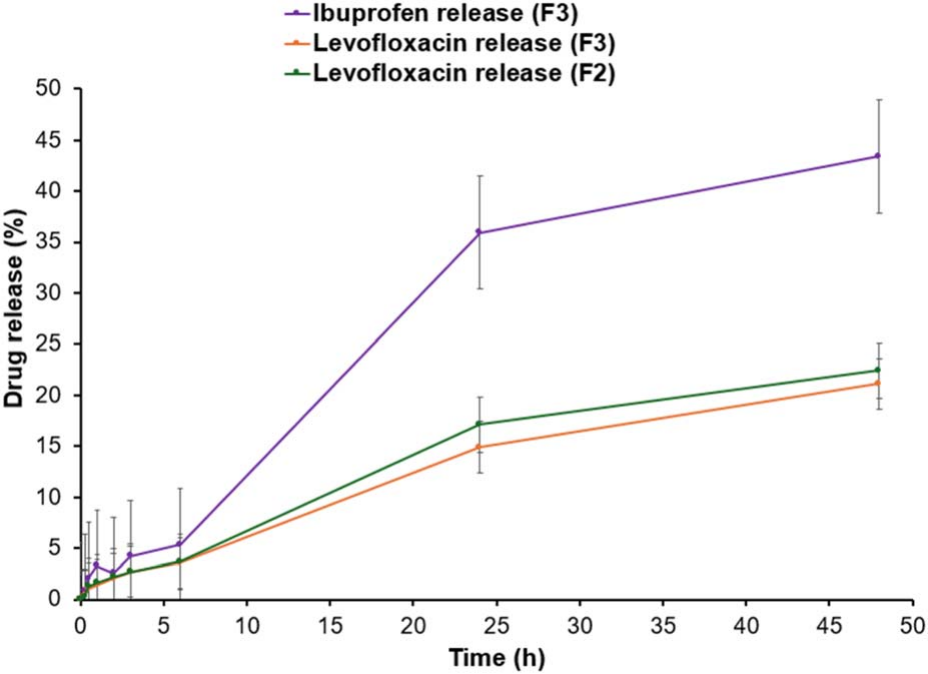
Comparative *in vitro* drug release profiles for levofloxacin from F2, and dual drug release from F3 for levofloxacin and Ibuprofen.

For drug-loaded formulations F2 and F3, the *in vitro* release of levofloxacin and ibuprofen was assessed in phosphate buffer saline pH 7.3 at 37 °C, over 48 hours (Fig. 7). The selected *in vitro* release conditions were designed to mimic the human physiological conditions (interstitial fluid/blood pH, body temperature), especially the potential applications of the sutures for internal organ and superficial surgeries. Drug release was primarily diffusion-controlled, with contributions from polymer relaxation (anomalous transport), according to the release data, which best fit the Korsmeyer–Peppas and Higuchi models for levofloxacin in both F2 and F3, indicating sustained matrix-driven release (Table 3). On the other hand, ibuprofen in F3 had weaker fits overall, with the highest *r*^2^ for Korsmeyer–Peppas, indicating that both surface desorption and diffusion contributed to its faster release, Fig. 7. According to the time-dependent release (Fig. 7), ibuprofen release from F3 over 48 hours was faster relative to levofloxacin release. In contrast, levofloxacin *in vitro* release was not affected by the presence of ibuprofen in the formulation since its release profiles are indicative of superimposition from both F2 and F3.

**Table 3:** Comparative *in vitro* release kinetic model to predict the release of loaded drugs from sutures

Formulation	Zero-order (*r*^2^)	First order (*r*^2^)	Higuchi (*r*^2^)	Korsmeyer–Peppas (*r*^2^)
F2 (levofloxacin)	0.9528	0.9603	0.9841	0.9886
F3 (levofloxacin)	0.9695	0.9764	0.9886	0.9943
F3 (ibuprofen)	0.9293	0.9436	0.9356	0.9764

The difference in the *in vitro* release of levofloxacin and ibuprofen from the same suture can be explained by their difference in physicochemical properties. Ibuprofen diffuses more easily through the PCL matrix due to its relatively low molecular weight of 206 Da,^35^ while levofloxacin migrates more slowly due to its larger molecular weight (398–740 Da), depending on salt form.^36^ Both compounds' release patterns are also influenced by their solubility profiles and ionisation behaviour. At physiological pH, ibuprofen is mostly in ionised form (p*K*_a_ 4.8–4.9), increasing its solubility in the phosphate buffer and speeding up diffusion. In contrast, levofloxacin is amphoteric, having p*K*_a_ values between 5.8 and 8.7,^37^ allowing it to interact ionically and/or form hydrogen bonds with PCL's carbonyl groups. Another possible reason could be that the drugs are distributed within the polymer matrix. Ibuprofen tends to partition closer to the polymer surface because it is more hydrophobic but easily ionisable. This allows for an initial burst release and a comparatively faster diffusion phase. Levofloxacin, on the other hand, interacts more strongly with the PCL backbone and becomes more evenly entrapped within the polymer, leading to a slower and more regulated release profile. These results are in line with earlier research that found that ibuprofen exhibits a biphasic release with an initial burst followed by faster diffusion,^38^ whereas levofloxacin release from PCL systems more gradually.^29,37,39^ Overall, the data show that the release kinetics are controlled by the physicochemical properties of the incorporated drugs, specifically their molecular weight, ionisation, solubility, and polymer affinity. As a result, levofloxacin has a slower release and ibuprofen has a relatively quick release, demonstrating the suitability of PCL sutures as a sustained delivery platform for these dual therapeutic agents.

The present study characterises the initial 48-hours *in vitro* release phase, which is particularly critical to the immediate postoperative period when the risk of microbial contamination is highest. However, these short-term data do not by themselves establish long-term antibacterial and/or anti-inflammatory efficacy. Similarly, our study established the baseline mechanical properties of fabricated drug-loaded sutures. However, a pertinent question is whether tensile strength changes during drug release and polymer degradation. As levofloxacin and ibuprofen diffuse out of the PCL matrix, the resulting porosity could theoretically reduce mechanical integrity. Additionally, PCL undergoes bulk erosion *via* hydrolytic cleavage of ester bonds, which may further affect tensile performance over extended periods. To address these critical questions, the suture is designed to be biodegradable, and some loss of mechanical strength over time is expected and acceptable, provided that the suture retains adequate tensile support throughout the critical wound healing period (typically 2–6 weeks for most surgical incisions). Relevant literature indicates that PCL degradation occurs over 12–24 months, an order of magnitude longer than the healing period.^40^ Furthermore, the mass loss and porosity development from drug diffusion are gradual and do not lead to premature failure during the early postoperative phase when mechanical support is most needed.

Therefore, although drug release initiates the formation of micropores, the impact on tensile performance during the clinically relevant timeframe is expected to be negligible. Nevertheless, we acknowledge that this interpretation is based on literature inference rather than direct experimental evidence from our study. Future work will include longitudinal mechanical testing of drug-eluting sutures at multiple and longer time points (*e.g.*, 1, 2, 4, 8, and 12 weeks) during *in vitro* degradation to directly quantify the relationship between cumulative drug release, porosity evolution, polymer erosion, and residual tensile strength.

### 3.8 Clinical relevance and study limitations

It is worthy to mention that one of the core rationales for our approach is to design a system that delivers dual agents locally and simultaneously to facilitate surgical wound healing and prevent complications. Thus, eliminating the need for additional oral and/or systemic antibiotics and other pharmacotherapeutic agents after stitches-related surgeries. Indeed, achieving 20% w/w drug loading embedded within the matrix in our suture as a combination therapy is another significant improvement to the available stitches-related surgical therapy, given that current commercial drug-coated sutures contain only a very small fraction of monotherapy, such as triclosan coated on the suture surface. Moreover, since the biphasic release pattern seen in this study closely resembles the temporal sequence of wound healing, it has direct clinical implications. Surgical wounds are particularly vulnerable to microbial contamination in the first 48 hours after surgery, and bacterial colonisation frequently starts in the first few hours after closure.^41^ This time frame is the most crucial for preventing SSIs. Levofloxacin's initial release from the sutures ensures that high local antibiotic concentrations are rapidly reached, exceeding the minimum inhibitory concentration (MIC) required to inhibit bacterial growth. By providing an antimicrobial barrier at the point of greatest vulnerability, such early drug availability lowers the risk of infection. Consequently, the localised nature of the antibiotic in our suture design is aimed at limiting systemic availability of the antibiotic in the body. Thus, this action would aid in preventing antimicrobial resistance.

The next stage of sustained release, which lasts for several days, corresponds with the inflammatory stage of wound healing. Neutrophil and macrophage infiltration, the release of pro-inflammatory cytokines, and clinical manifestations such as pain, redness, and swelling are characteristic of this stage.^42^ A more favourable environment for tissue repair is created while local inflammation and discomfort are decreased by the anti-inflammatory effects of ibuprofen's gradual release during this time. Thus, ibuprofen and levofloxacin would have complementary dual actions, one addressing microbial contamination and the other reducing excessive inflammation and pain.

Wounds usually enter the proliferative and early remodelling phases after the first week, which are marked by angiogenesis, collagen deposition, fibroblast proliferation, and progressive tissue strengthening. Remaining antimicrobial coverage may still help prevent late-onset complications, especially in immunocompromised patients, even though the risk of infection is lower at this point. Furthermore, prolonged inflammatory responses that might otherwise postpone tissue regeneration may be modulated by the continuous release of ibuprofen.^43^ Further biological validation and degradation studies are required prior to clinical translation.

An important characteristic of sutures is their tensile strength, or breaking strength, which is primarily determined by the suture's diameter. Suture sizes are identified numerically based on their diameter. Sutures are labelled from 0 to 10, with #10 representing the largest diameter. Thinner sutures are designated by increasing numbers of zeros, ranging from 1–0 to 12–0, with 12–0 having the smallest diameter and the lowest tensile strength. The diameter difference between adjacent suture sizes typically ranges from approximately 0.01 to 0.05 mm.^44^ In this study, we have all three fabricated sutures with USP classifications, diameter 2–0 and 3–0. Clinically, sutures of these sizes find wide usage in general surgery, gastrointestinal surgery, soft-tissue approximation, ligation, and fascia closure, where moderate support is needed during the healing process.^45^ The diameter provides adequate breaking strength to resist physiological stress.

Additionally, our approach supports the recent “Treating conflict wounds” challenge issued by UK Defence Innovation (UKDI), which falls under their priority interests in battlefield injury care. Seeking alternative solutions to prevent wound infection and tissue death in harsh environments where medical evacuation is delayed. Our multi-drug-releasing sutures demonstrate early and sustained release of antimicrobial, anti-inflammatory and pain-relieving agents, meeting the needs of conflict wounds for strong, easy-to-carry treatments. This directly addresses the serious issue of combat-related wound infections.^46^ Other than human surgical applications, 2–0 and 3–0 sutures are also often utilised in veterinary practices to close muscle, fascia, and subcutaneous tissues in small and medium animals.^47^

Some limitations must be acknowledged from the current study. Specifically, the lack of *in vitro* antimicrobial testing, cytotoxicity evaluation, knot security, and *in vivo* performance assessments restrict this study. Future studies will include antibacterial testing against clinically relevant pathogens such as *Staphylococcus aureus*, *Escherichia coli*, and *Pseudomonas aeruginosa*, with benchmarking against commercially available triclosan-coated sutures such as Vicryl Plus®. In addition, anti-inflammatory and wound-healing efficacy should be evaluated using appropriate *in vitro* and *in vivo* models.

## 4. Conclusion

In this work, we have successfully prepared biodegradable PCL sutures incorporating clinically relevant antibiotic and anti-inflammatory therapeutic agents (levofloxacin and ibuprofen) through a solvent-free hot-melt extrusion technique. The drugs were stably embedded in the PCL matrix without compromising suture integrity, as evidenced by molecular dispersion and good drug compatibility within the polymer matrix. An advantageous dual-release pattern was observed: Ibuprofen is released earlier to be able to control inflammation, while levofloxacin releases more slowly for sustaining antimicrobial prophylaxis. The current study uses a dual-drug approach with clinically relevant drugs, whereas existing commercial sutures deliver only one antimicrobial agent that barely serves the SSI's protective purposes. Also, the use of hot-melt extrusion eliminates the organic solvents required in coating methods, leading to a process that is safer, cleaner, and more sustainable and scalable. This work follows the recent trends in pharmaceutical engineering dealing with the development of a new generation of implantable devices characterised by multifunctional capabilities. An improvement in mechanical strength and possibly modifications in release profiles can be achieved by optimising drug ratios and/or polymer blends. Although PCL is biodegradable, the exact degradation time of the present suture geometry requires an *in vivo* evaluation, as degradation depends on filament dimensions, implantation site, and local physiological conditions. Future studies will assess whether mechanical support is retained until complete wound healing is achieved. Considering the limitations noted, this study offers a proof-of-concept and a viable foundation for developing next-generation sutures that would enhance postoperative wound healing. Thus, we present a distinctive and clinically applicable strategy to reduce postoperative complications, including prophylaxis against SSIs, through an innovative multifunctional suture design.

## Author contributions

Garba Mohammed Khalid: conceptualisation, project supervision, resources, writing – original draft review & editing, project administration, methodology, visualisation, formal analysis, data curation. Néel Anil Chaudhary: writing – original draft, visualisation, validation, methodology, formal analysis. Nura Hassan: investigation, methodology. Lubabah Rahman: investigation, methodology. Shahnaz Kashif: investigation, review and editing. Gunjan V. Jahakar: investigation, review and editing. Mouhamad Khoder: methodology, review and editing.

## Conflicts of interest

There are no conflicts to declare.

## Data Availability

All the data used to support this study are presented within the article, and no additional source data are required.
